# Case report: Germline *POT1* mutation in a patient with GIST and lung adenocarcinoma

**DOI:** 10.3389/fonc.2024.1419739

**Published:** 2024-08-02

**Authors:** Stefania Martino, Simona De Summa, Brunella Pilato, Maria Digennaro, Letizia Laera, Stefania Tommasi, Margherita Patruno

**Affiliations:** ^1^ Center for Study of Heredo-Familial Tumors, IRCCS Istituto Tumori “Giovanni Paolo II”, Bari, Italy; ^2^ Molecular Diagnostics and Pharmacogenetics Unit, IRCCS Istituto Tumori “Giovanni Paolo II”, Bari, Italy; ^3^ Department of Oncology, “F. Miulli” General Regional Hospital, Acquaviva Delle Fonti, Italy

**Keywords:** tumor predisposition syndrome, lung adenocarcinoma, GIST, POT1, case report

## Abstract

The gene *protection of telomere 1* (*POT1*) is involved in telomere maintenance and stability and plays a crucial role in the preservation of genomic stability. *POT1* is considered a high-penetrance melanoma susceptibility gene; however, the number of cancer types associated with the pathogenic germline variants of *POT1* is gradually increasing, including chronic lymphocytic leukemia (CLL), angiosarcomas, and gliomas, even though many associations are still elusive. Here, we reported a case of a 60-year-old man who showed early-onset multiple neoplasms, including multiple melanomas, gastrointestinal stromal tumor (GIST), and lung adenocarcinoma. Next-generation sequencing (NGS) analyses revealed a germline heterozygous pathogenic variant in the *POT1* gene. Notably, GIST and lung adenocarcinoma were not previously reported in association with the *POT1* germline variant. Lung cancer susceptibility syndrome is very rare and the actual knowledge is limited to a few genes although major genetic factors are unidentified. Recently, genome-wide association studies (GWAS) have pointed out an association between *POT1* variants and lung cancer. This case report highlights the clinical relevance of *POT1* alterations, particularly their potential involvement in lung cancer. It also suggests that *POT1* testing may be warranted in patients with familial cancer syndrome, particularly those with a history of melanoma and other solid tumors.

## 1 Introduction

The gene *protection of telomere 1* (*POT1*) encodes a crucial component of the human shelterin complex, involved in the regulation and preservation of telomeric ends (1). Deleterious genetic variants in *POT1* are involved in telomere biology disorders (TBD), characterized by abnormal telomeric length (2). Indeed, *POT1* mutations may generate two opposite phenotypes of telomere elongation or telomere shortening: the first one is associated with neoplastic predisposition, and the second one is involved in telomeropathies (3). Germline loss-of-function (LOF) variants in *POT1* are associated with a rare oncological predisposition syndrome, known as POT1-tumor predisposition syndrome (POT1-TPD), with no more than 100 affected families currently reported (4). POT1-TPD is inherited with an autosomal dominant pattern, typically manifesting in adulthood. However, genetic anticipation and gradual lowering of the age of onset across successive generations have been observed (5). POT1-TPD is associated with an increased lifetime risk of multiple melanomas (6), chronic lymphocytic leukemia (CLL), angiosarcoma (7, 8), and glioma (9). Despite its rarity, *POT1* mutations have been also reported in various kinds of neoplasms including colorectal cancer (10), thyroid cancer (11), uveal melanoma (12), and myeloma (13). Definitive associations, however, remain elusive due to the lack of consistent data. Here, we report a unique case of a male patient diagnosed with POT1-TPD, subsequently found to be manifesting gastrointestinal stromal tumor (GIST) and lung adenocarcinoma. To the best of our knowledge, this is the first reported case of GIST and lung adenocarcinoma associated with a *POT1* germline variant. This case represents an example suggesting the clinical relevance of *POT1* alterations and their involvement in a broad range of cancer types and indicates the possibility that management approaches should be evaluated for mutation carriers.

## 2 Case description

**Figure 1:**
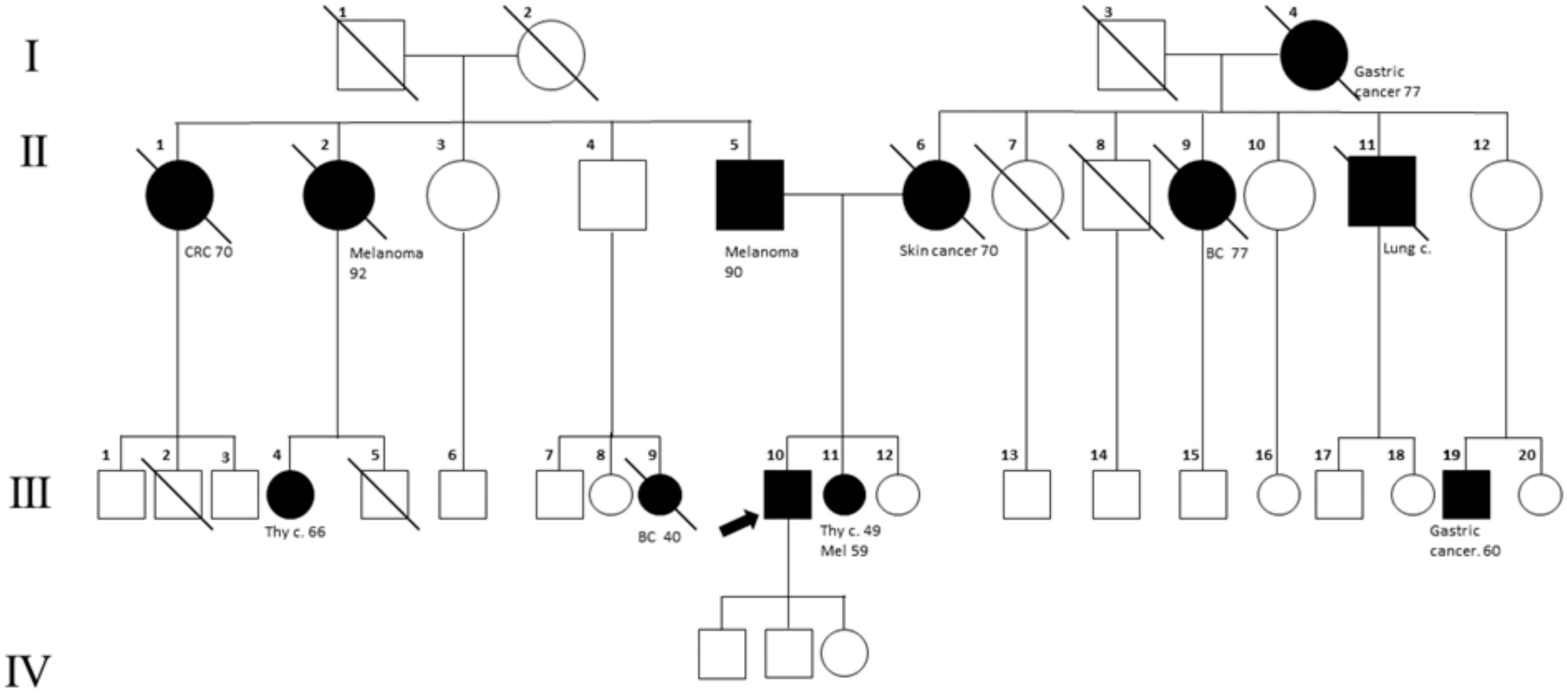
Family tree. The proband is indicated with an arrow (III_10). Melanoma cases are reported in the father (II-5), sister (III-11), and paternal aunt (II-2). The mother (II-6) has been reported to have a not well-defined “skin cancer.” In the family, other cancer types are present: breast cancer (BC; II-9, III-9), lung cancer (III-11), gastric cancer (III-19, I-4), colorectal cancer (CRC, II-1), and thyroid cancer (III-11, III-4).

**Figure 2:**
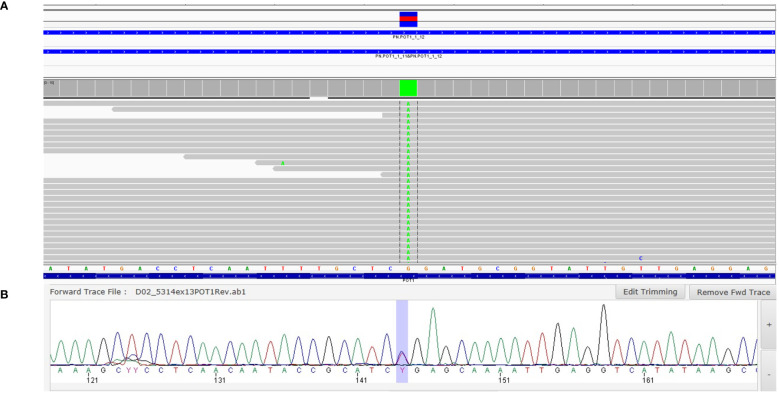
IGV screenshot and Sanger sequencing. On the top, the IGV screenshot is depicted **(A)**, showing the variant R363* in POT1, in a heterozygous state, identified in the NGS analyses. On the bottom, an electropherogram of Sanger sequencing confirms the NGS results **(B)**.

**Figure 3:**
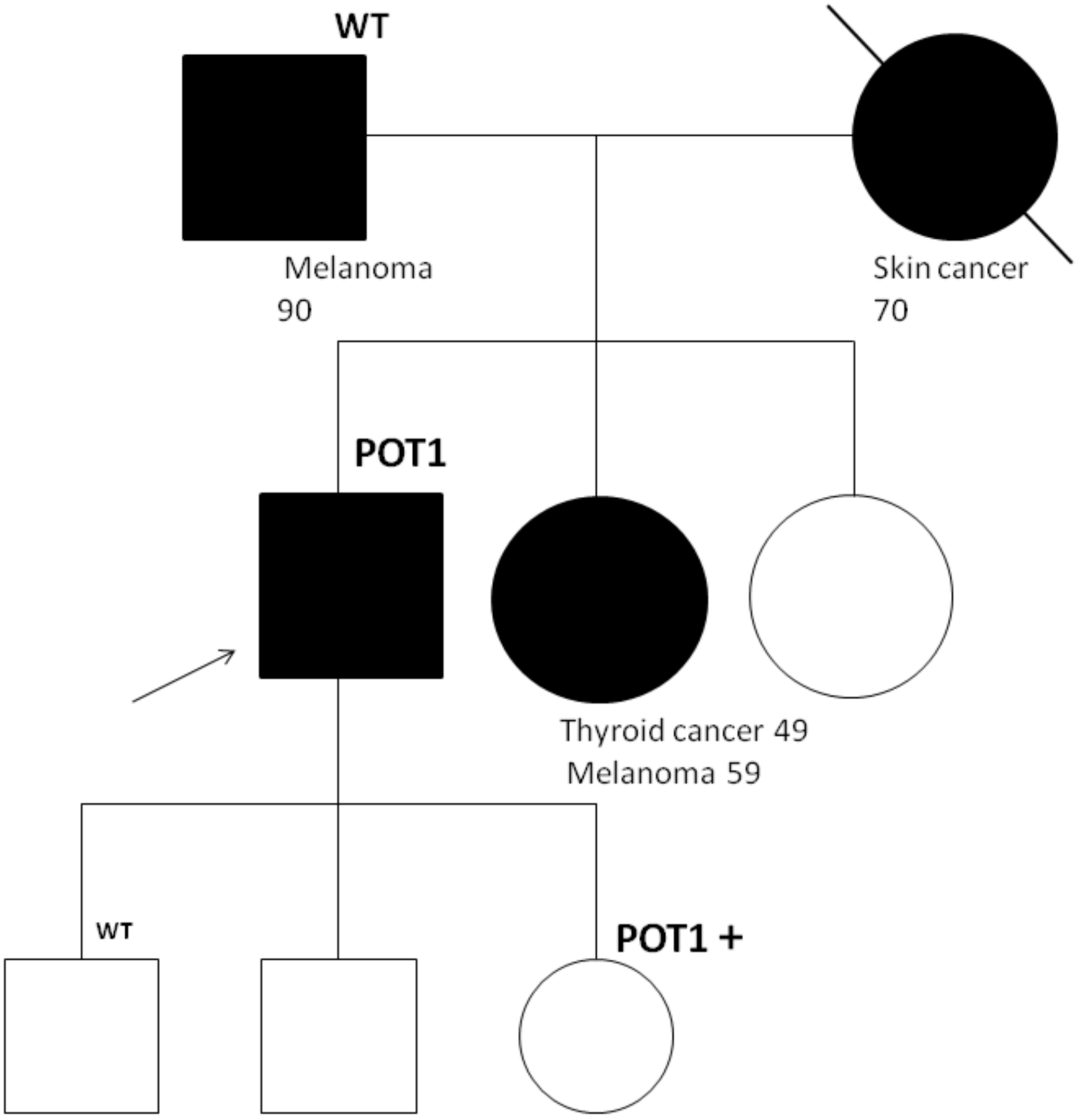
Segregation analysis for the *POT1* variant R363*. The proband is indicated with an arrow. In the father, daughter, and son, segregation analysis by Sanger sequencing was performed. The daughter, who was positive for the *POT1* variant, underwent exportation of two basal cell carcinoma after the test.

A 60-year-old man, who is a non-smoker, was referred by an oncologist to our unit for oncogenic counseling, prompted by a suspicion of an oncological predisposition syndrome. His medical history (**Table 1**) outlined a series of neoplastic events, beginning with surgical excision of different suspected pigmented lesions, one of which was classified as melanoma *in situ*. He also underwent a hemicolectomy for a mesenchymal neoplasm identified as GIST in the left colon, and at that time, a right adrenal adenoma was identified during an MRI exam. Another surgical intervention involved the removal of a metastatic intramedullary neoplasm at D-10 and D-11 vertebral levels. The metastasis originated from melanoma even though the primitive lesion was not identified. The patient had recently been diagnosed with papillary lung adenocarcinoma. Examination of familial history also pointed to a possible genetic syndrome of oncological predisposition (**Figure 1**), given the cases of melanoma in the patient’s sister (III-11), paternal aunt (II-2), and father (II-5), coupled with an indistinct skin condition in the mother (II-6), referred to as “skin tumors” that had not been well characterized. Given the clinical suspicion of the presence of a genetic risk factor predisposing to melanoma, genetic analysis was indicated. Genomic DNA was extracted from the proband’s peripheral blood, and analysis was conducted using next-generation sequencing (NGS). The targeted custom panel was used to examine six hereditary melanoma susceptibility genes (*CDKN2A*, *MITF*, *MC1R*, *CDK4*, *POT1*, and *BAP1*). Libraries were prepared with the Ion AmpliSeq Library Kit Plus and were sequenced with Ion Torrent S5 (Thermo Fisher, Waltham, USA). Variant calling was performed through the Torrent Variant Caller after alignment using GRCh37 as a reference. The analysis revealed the presence of the heterozygous pathogenic variant c.1087 C>T; p. (Arg363*)(rs756198077), in the *POT1* gene (NM_015450.3), with the GRCh37 coordinate chr7:124482937. The alteration was confirmed by Sanger sequencing (**Figure 2**). This variant is present on the gnomAD database, with a maximum subpopulation frequency of 0.0047% in the European (non-Finnish) population (7–124482937-G-A, gnomAD v2.1.1, gnomad.broadinstitute.com). Loss of function is an established mechanism of the disease in the *POT1* gene. Fifty-two nonsense variants are reported on the ClinVar database (ncbi.nlm.gov/clinvar/), most of which (51/52) were classified as likely pathogenic/pathogenic. Indeed, ClinVar contains an entry for the identified variant (Variation ID: 475019). This finding led to the diagnosis of POT1-TPD. In the absence of official guidelines for POT1-TPD, the only surveillance plan suggested is very similar to that for the Li–Fraumeni-like syndrome (14), due to the comparable associated phenotypical spectra. The recommended surveillance, indicated for the patient, includes dermatological checkup every 6 months, routine complete blood count (CBC) with differential analysis, lymph node palpation, and annual brain and whole-body MRI. Since beginning at age 18 years old. We recommended expanding the genetic analysis to the family (**Figure 3**), and relatives were tested by Sanger sequencing. Segregation analysis was performed on the father, daughter, and son. The daughter (IV-3), 32 years old, is the sole family member who tested positive for the *POT1* variant (**Figure 3**) and had two suspected lesions that were removed from the right side and the back after both were identified as basal cell carcinoma. The same surveillance protocol was recommended, given the oncological clinical history of her father.

**Table 1:** The patient’s clinical history.

**Year**	**Age of diagnosis**	**Operation/exam**	**Location**	**Type**
2007	45	Excision pigmented lesion	Back	Melanoma *in situ*
2008	46	Excision pigmented lesion	Right pectoral region	Dysplastic compound nevus
2015	53	Excision pigmented lesion	Left thigh	Cutaneous junctional nevus
2016	54	Hemicolectomy	Right colon	GIST
2016	54	MRI	Abdomen	Right adrenal adenoma
2018	56	Excision intramedullary neoformation	D-10–11	Melanoma
2022	60	Nodule excision	Right Lung	Lung papillary adenocarcinoma

## 3 Discussion

In this paper, we report a 60-year-old male patient affected by a series of malignancies including multiple melanomas, GIST, adrenal adenoma, and lung adenocarcinoma, with a familial history marked by numerous cases of melanoma. Genetic analysis identified the pathogenic variant p.R363* in the *POT1* gene. The variant R363* was previously identified in cases of colorectal cancer (9). LOF mutations in *POT1* are associated with telomeric lengthening (5), increased longevity, and the promotion of carcinogenesis (4, 15). Our case, notably featuring GIST in association with a *POT1* mutation, contributes additional insights to the existing literature. Furthermore, given that major genetic factors for lung cancer predisposition are still to be identified, our study provides the first reported clinical evidence supporting previous genome-wide association studies (16, 17) that indicate a potential association between *POT1* variants and lung cancer (18). Notably, a pan-cancer study on 62,368 tumors revealed that deleterious *POT1* variants are present in approximately 5% of non-small cell lung cancers (19). Furthermore, an observational study on 95,568 individuals of a general population underscored the correlation between longer telomeres and increased risk of both melanoma and lung cancer (18). In light of current knowledge, the Li–Fraumeni-like surveillance protocol, described by Henry et al. (14), is the only available proposal at this time. However, the discussion about the suitability of this protocol is still open, considering the burden of oversurveillance in *POT1* variant carriers. It is important to specify that surveillance should be modulated on the emerging phenotypic spectrum of POT1-TPD and the individual’s personal family history (14). However, in this case, we decided to recommend the Li–Fraumeni-like surveillance protocol to *POT1* variant carriers, especially for the proband’s daughter given the personal clinical history of the proband and the lack of known environmental risk factors. Indeed, MRI is reliable in the detection of both GIST lesion (20) and early lung cancer (21), and despite its limitations, it is considered a safe alternative to TC, especially in the context of oncological screening, in healthy subjects, which should be reiterated for a long period of time. In exploring this unique case, we aim to contribute valuable insights into the clinical relevance of *POT1* mutations, particularly given the scarcity of available data and the current lack of a definition of the complete phenotypical spectrum associated with *POT1*. Our findings also prompt considerations for tailored management approaches for individuals carrying *POT1* mutations, suggesting the need for vigilant surveillance and genetic counseling.

## Data availability statement

The raw data supporting the conclusions of this article will be made available by the authors, without undue reservation.

## Ethics statement

The description of a case report does not require ethical approval. The studies were conducted in accordance with the local legislation and institutional requirements. The participants provided their written informed consent to participate in this study. Written informed consent was obtained from the individual(s) for the publication of any potentially identifiable images or data included in this article.

## Author contributions

SM: Conceptualization, Visualization, Writing – original draft, Writing – review & editing. SD: Data curation, Formal analysis, Investigation, Visualization, Writing – review & editing. BP: Investigation, Validation, Visualization, Writing – review & editing. MD: Visualization, Writing – review & editing. LL: Investigation, Visualization, Writing – review & editing. ST: Visualization, Writing – review & editing. MP: Conceptualization, Funding acquisition, Supervision, Visualization, Writing – review & editing.
